# Promoting Physical Activity and Reducing Sedentary Behavior to Prevent Chronic Diseases during the COVID Pandemic and Beyond

**DOI:** 10.3390/jcm11164666

**Published:** 2022-08-10

**Authors:** Zan Gao, Jung Eun Lee

**Affiliations:** 1 School of Kinesiology, University of Minnesota, Minneapolis, MN 55455, USA; 2 Department of Applied Human Sciences, University of Minnesota, Duluth, MN 55812, USA

Physical activity is defined as any physical motion produced by skeletal muscle that causes a notable increase in energy used compared to at rest [1]. As known, decreased physical activity participation and increased sedentary behavior increase the risks of developing chronic diseases such as hypertension and diabetes among various populations, particularly at the era of the COVID-19 pandemic [2,3,4,5,6,7]. Regular participation in physical activity and reducing sedentary behavior play a significant role in health promotion and disease prevention across a person’s lifespan. More specifically, moderate-to-vigorous physical activity helps build and maintain healthy bones and muscles; reduces the risk of developing obesity and chronic diseases such as cardiovascular disease; and diminishes symptoms of depression and anxiety, thereby promoting cardiorespiratory fitness and psychological well-being [8]. However, globally, 81% of adolescents aged 11–17 years and approximately 23% of adults aged 18 and over were insufficiently physically active [9]. In fact, physical inactivity is one of the ten leading risk factors for global mortality, which is on the rise in many countries, adding to the burden of non-communicable diseases (e.g., cardiovascular diseases, cancers, and diabetes) and affecting general health worldwide. Therefore, the study of promoting physical activity and reducing sedentary behavior to prevent chronic diseases has become an emerging trend in the field, as an increasing number of researchers have conducted investigations in this area in recent decades. In response, we invited investigators to contribute 15 original research articles and review articles that could stimulate the continuing efforts to understand the relationships between physical activity, sedentary behavior, and health outcomes among various populations. In this Special Issue, we were particularly interested in articles examining the effects of physical activity programs on health promotion and disease prevention, as well as correlates and determinants of physical activity and sedentary behavior across lifespan during the COVID-19 pandemic and beyond.

Changes in physical activity behaviors and determinants in the era of the COVID-19 pandemic have become a public health concern over the past few years [10,11,12,13,14]. In Wang et al.’s study [15], 1028 Chinese adults were recruited via probability sampling to examine any changes in participants’ physical activity behavior before and during the COVID-19 pandemic. The researchers found that, after seven months of the pandemic outbreak, Chinese adults’ weekly moderate-to-vigorous physical activity significantly decreased from 139 min to 120 min, especially among females and rural populations. It was also revealed that over 46% of the sample did not meet the physical activity guidelines before or during the pandemic. Additionally, further inquiry of those who remained highly active demonstrated that the main predictors were their intrinsic motivation, the number of sport skills acquired, and their participation in sports organizations. 

Jung et al. [16] investigated the association between participants’ changes in work schedule and their health behavior changes using the data from the Korea Labor and Income Panel Survey prior to the pandemic. A generalized estimating equation was used to analyze health behavior changes after adjusting for age, sex, education level, occupational classification, income level, and self-rated health status. They found that changes from fixed daytime to shift schedule and from shift to fixed daytime work contributed to 670 and 739 person years, respectively. Additionally, the researchers found that workers who are continuously exposed to a shift work schedule and those whose worked a schedule changed from regular daytime to shift work were vulnerable to adopting unhealthy lifestyle habits, such as smoking and drinking. 

In another cross-sectional study, Wärnberg and colleagues [17] investigated the relationship between Spanish children’s (8 to 16 years old) screen time, physical activity behaviors, parents’ education level, and children’s adherence to the Mediterranean dietary pattern. Results revealed that a greater amount of children’s screen time and lower education levels in parents were associated with children’s poorer adherence to the Mediterranean diet. In other words, children consumed lower amounts of fruits, vegetables, fish, legumes, and nuts, while consuming more fast food and sweets. The researchers concluded that, among children, passive screen time should be used in a limited and responsible manner, especially those with parents with a lower education level.

From another methodological perspective, Gao and colleagues [18] reviewed different methodologies for analyzing wrist-worn accelerometer data and offered cutting edge, but appropriate, analysis plans for wrist-worn accelerometer data in the assessment of physical behavior (e.g., physical activity and sedentary behavior). They discussed various methods of processing these data (e.g., cut points, steps per minute, machine learning), and the opportunities, challenges, and directions for future studies in this area of inquiry. This is the most comprehensive review paper concerning the analysis and interpretation of free-living physical activity data derived from wrist-worn accelerometers, aiming to help establish a blueprint for processing wrist-derived accelerometer data. 

**Cardiorespiratory Health**

From as early as the late 1950s, countless studies have investigated the relationships between physical activity and cardiovascular health. Panels formed by experts convened by national and international organizations—as well as the 1996 U.S. surgeon General’s report on physical activity and health—have reinforced the scientific evidence that physical activity positively affects various aspects of cardiovascular health [19]. Due to more than half a century of epidemiological studies, it is now generally undisputed that physically active people present lower risks for developing coronary heart disease compared to those who are inactive, and that physical activity patterns and cardiorespiratory fitness levels are associated with greater health outcomes [20,21,22].

In a meta-analysis of this Special Issue, Sašek and colleagues [23] reported that participants’ cardiorespiratory-related functional performance (i.e., walking test, timed-up-and-go-test) improved as early as three to six months after lower limb joint arthroplasty, and an increase in moderate-to-vigorous physical activity (MVPA) and step counts were observed after 6 months, with greater increased MVPA occurring after 12 months. However, participants’ sedentary behavior did not improve at six to nine months, and their physical activity levels remained lower than their healthy counterparts 12 months post-surgery. The researchers suggested more research on rehabilitation programs to improve cardiorespiratory health using innovative technology with behavioral therapy.

**Metabolic Health**

Scientific evidence strongly suggests that regular physical activity increases metabolic health by 30–40% in individuals who are at minimum moderately active compared to sedentary individuals, with the metabolic health benefits being equally applicable to a wide range of populations. Generally, the physical activity related adaptations that benefit metabolic health are the same as those in cardiorespiratory adaptation to physical activity, including increased high-density lipoprotein levels, lower low-density lipoprotein levels [24], and lower triglyceride levels [25]. Furthermore, both aerobic and resistance exercise increase the abundance of glucose transporter protein type 4 (GLUT4) and blood glucose uptake [26]; with an increase in GLUT4 levels due to physical activity, insulin resistance was prevented [27], while improvements in insulin sensitivity, which is key in preventing and treating diabetes, were observed [28]. 

Current evidence also suggests that consistent physical activity is highly associated with improved glucose tolerance [29], increased protein synthesis rates and amino acid uptake by skeletal muscle [30], as well as improved body composition [31,32]. Overall, significant adaptations result from responses to physical activity and exercise—by improving metabolic health, individuals can reduce risks of chronic disease and improve their quality of life. 

In this Special Issue, Neto and colleagues [33] investigated the relationships between specific sedentary behavior, TV viewing with physical activity, and cardiometabolic risk factors among 2155 European adults. Individuals with high TV viewing were associated with higher amounts and an inappropriate distribution of adipose tissue, independent of physical activity levels. That is, those with a high level of TV viewing and high physical activity level demonstrated a 1.03 higher body mass index (BMI), 2.42 cm greater waist circumference, and 2.4% higher body fact percentage than the reference group. The results indicate the health risk of sedentary behavior even among those with a high level of physical activity.

In the study of Rapisarda et al. [34], thirty-eight healthcare workers with at least one cardiovascular risk factor participated in a program that included their diet, physical activity, metabolic and anthropometric measures being assessed at baseline, after 6 months, and after 12 months. The multidisciplinary intervention included sports activities, counseling, clinical and instrumental evaluation, diet and physical activity monitoring, and an individualized Mediterranean diet program to focus on improving lifestyle. At 12 months, participants’ metabolic measures such as blood glucose, blood pressure, BMI, and total cholesterol were reduced, while their work performance and perception of their body image improved. The researchers highlighted the importance of using a multidisciplinary intervention and the role of occupational medicine in improving healthcare workers’ health.

In addition, Cao and colleagues [35] examined the combined effects of progressive aerobic exercise and high-intensity interval training on fat reduction among 84 obese Chinese adults. The intervention group performed exercise three times per week for seven weeks, while the control group were instructed to continue their normal activities. After seven weeks, they found that the intervention group demonstrated significant increases in fat oxidation at rest, and showed a significant decrease in BMI, body fat percentage, visceral fat area, and total cholesterol at maximal oxygen intake. 

**Musculoskeletal Health**

Regular physical activity participation was also found to be an effective measure for improving musculoskeletal functioning [36]. Most healthy individuals were observed to have improved muscular strength and endurance with only moderate intensity physical activities, such as weight bearing and stair climbing. 

For those who performed interval training which combined resistance training with aerobic exercise, a VO_2_ max increase (5% change) was observed; even sedentary individuals could increase their VO_2_ max by 3% just by participating in resistance training alone [37]. When physical activity programs are combined with resistance training, both strength and explosive power were also observed to have increased over time [38,39]. The improved ability to recruit more motor units (i.e., motor neurons and the muscle fibers it innervates) by the body, the growth of individual muscle fibers, the increase in numbers of anaerobic enzymes, and the increased anaerobic energy reserves all promote musculoskeletal strengthening. All of the aforementioned health benefits are essential for engaging in short-term and high-intensity musculoskeletal movements. 

Clinically significant improvements in ligament and tendon strength, as well as increased collagen content, may reduce the risk of injury in individuals for any age group; all of these benefits are the result of connective tissue modifications caused by placing stress on specific muscles during musculoskeletal activities. Meanwhile, positive hormonal changes that lead to bone remodeling can be triggered by resistance training, which can increase bone mineral density in some and delay bone loss in others. Both are important for those at risk for osteoporosis, especially women. 

Studies in exercise physiology revealed a clear increase in lean muscle mass due to the improved muscle mass and quantity that is brought on by regular physical activity and musculoskeletal strength training, which also reduces body fat and helps in losing or maintaining weight [40]. Interestingly, the amount of physiological adaptation associated with each of the benefits of physical activity reviewed to date is dose dependent, meaning that lower doses of physical activity have smaller physiological effects and alterations compared to higher doses [41]. Thus, it is crucial to keep this dose–response relationship in mind when reviewing the literature on physical activity interventions or when developing interventions.

In this Special Issue, Żywień and colleagues [42] estimated risk of low back pain by comparing the pressure pain threshold of soft tissue and the angles of the spine in young white-collar workers. The results indicated that pressure pain thresholds and the angle of the spine in the sitting position were associated with mild low back pain in female subjects. In addition, the mild low back pain was related to the following in male participants: angles of torso; the lumbosacral spine in the corrected sitting position; and body mass index. The researchers also revealed that workers with low-intensity, non-specific back pain had similar pressure pain thresholds of the soft tissue to the asymptomatic participants. It was suggested that sedentary workers should be encouraged to self-correct posture and receive specific postural training to increase their awareness in postural control capacity to prevent and alleviate back pain.

Navarro-Patón and colleagues [43] examined the motor competence of 28 preschoolers (4–6-year-olds) with developmental coordination disorder in Spain. The intervention consisted of weekly physical education sessions led by a movement specialist that concentrated on developing children’s motor skills such as manual dexterity, aiming, catching, and balancing. The results indicated that there was a significant improvement in motor skills (i.e., manual dexterity, aiming and catching, balancing, and total test score) for children in the intervention group, as well as significant group differences between intervention and control groups. The findings demonstrate the effectiveness of a structured motor skill curricular program compared to traditional physical education program in young children with movement disorder. 

Additionally, it is important to investigate how correlates of physical activity (e.g., motor skill competence) among preschool children change over time. Ryu et al. [44] used a cross-lagged panel model to examine bidirectional relationships between motor skill competence, perceived competence, and physical activity among 61 preschool children. Bidirectional relationships between all the variables of interests were not observed in preschool children; however, there were some notable gender differences in each cross-lagged model. Although baseline motor skill competence was a significant predictor of children’s post-intervention motor skill competence, the predictability of baseline motor skill competence for post-intervention physical activity was seen only in girls. The association between baseline motor skill competence and post-intervention perceived competence was only observed in boys.

**Mental Health**

Mental disorders have long-term effects on an individual’s mood or emotions, personality, cognition, and perception, and consequently pose significant implications for public health [45]. Common mental disorders or problems associated with physical activity are listed by the Physical Activity Guidelines Advisory Committee [19] including mood disorders, anxiety disorders, psychological distress, low self-esteem, age-related cognitive decline, and diet or exercise-related disorders. Among the most frequently examined mental health outcomes associated with physical activity, investigations included mood, self-efficacy, self-esteem, and cognitive function—of these outcomes, affective (mood) and anxiety disorders were reported most frequently [46,47,48]. 

Current scientific evidence supports that regular physical activity and exercise reduces the risk and symptoms of depression [49], anxiety disorders [50], psychological distress [51], and age-related decline in cognitive function [52]. Furthermore, improvements in positive affect, general mental health [53], and self-esteem [54] were also observed. Overall, sedentary individuals were twice as likely to experience symptoms of depression and anxiety compared to active individuals. The general consensus is that people who engage in high levels of physical activity or have greater cardiorespiratory fitness tend to have better mood (greater positive affect and lower negative affect), higher self-esteem, more confidence in their ability to perform tasks requiring physical activity, and greater cognitive functioning. It should be noted that light physical activity (e.g., performing activities under daily living) may not be enough to induce a significant physiological response. Thus, moderate to vigorous levels of intensity during physical activity is recommended to elicit the physiological stimulation necessary for promoting mental health [41]. 

However, while physical activity can improve mental health, it has not been shown to be effective in the treatment of mental health disorders, particularly in the era of the pandemic [55,56]. That is, there is little research on the role physical activity may potentially play in the prevention of mental health disorders; in fact, the available literature examining individuals with mental health disorders such as depression and anxiety represents the best evidence available on the promotion of physical activity to prevent mental health disorders. As for literature on the psychological effects of regular physical activity on individuals with relatively good health or those with other mental disorders, such as sleep and eating disorders, schizophrenia, dementia, personality disorders, and substance-related disorders, it is unfortunately less clear. As such, more research is needed to further explore how physical activity affects mental health. 

In this Special Issue, Chen et al. [57] assessed the feasibility of establishing a 12-week (45 min per session) Tai Chi program (Sun Style Tai Chi) in a 75 bed-assisted living facility and evaluated the potential of the Tai Chi program to improve the fear of falling, functional mobility, and quality of life (e.g., depression, anxiety). This quality improvement project suggested that Tai Chi is a feasible exercise that might have the potential to reduce the risk of falls in older adults, and the program was well accepted with no serious or other adverse events reported. 

Through a comprehensive meta-analysis, Swora et al. [58] found a consistent pattern of associations between higher levels of physical activity and lower positive, negative, and general psychopathology symptoms in people with schizophrenia and those with other psychotic disorders. In another systematic review, Lee et al. [59] investigated the role of Pokémon GO, an augmented mobile app game, on players’ physical activity, as well as psychological and social outcomes. They found that players had significantly greater daily steps and number of days spent in moderate physical activity than non-players. In addition, they found that the game reduced players’ negative affect just after playing, as well as their neurotic personality trait and psychological distress over a few months of playing the game. On the contrary, their social interaction and sense of belonging improved. In terms of cognitive outcomes, the game also had a positive influence on adolescents’ selective attention and concentration, and young adults’ verbal working memory.

In conclusion, physical activity is well-documented to have many benefits and physiological effects for people. Most widely recognized are its effects on the cardiopulmonary and musculoskeletal systems, although the benefits to the body’s metabolic system are also notable. These physiological effects have been observed in individuals belonging to all races/ethnicities, genders, and ages. Moreover, the alleviation of anxiety and depressive symptoms, as well as improvements in mood, self-esteem, cognitive function, and general mental health, are all promising benefits of physical activity. 
